# Harnessing peer mentorship as a tool to turn human resource for health brain drain into brain gain: a case study of a Nigerian peer-mentored research group

**DOI:** 10.1186/s12960-024-00932-x

**Published:** 2024-06-27

**Authors:** Patience Toyin-Thomas, Oghenebrume Wariri, Paul Ikhurionan

**Affiliations:** 1 The Department of Pediatrics, Geisel School of Medicine, Dartmouth College, Hanover, NH United States of America; 2 The Dartmouth Institute for Health Policy and Clinical Practice, Lebanon, NH United States of America; 3 Department of Infectious Disease Epidemiology, London School of Hygiene and Tropical Medicine, London, United Kingdom; 4 Vaccines and Immunity Theme, Medical Research Council Unit The Gambia at London School of Hygiene and Tropical Medicine, Banjul, The Gambia; 5 Department of Child Health, University of Benin Teaching Hospital, Benin, Nigeria

**Keywords:** Peer mentorship, Research capacity development, Human resources for health, Low- and middle-income countries, Brain-drain, Brain-gain

## Abstract

**Background:**

Peer mentorship can be a potential tool to reduce the disparities in health research capacity between high- and low- and middle-income countries. This case study describes the potential of peer mentorship to tackle two critical issues: bridging health research capacity of doctors from low- and middle-income countries (LMICs) and the transformation of human resource for health brain drain into "brain gain".

**Case presentation:**

In 2021, a virtual peer mentorship group was established by 16 alumni of the University of Benin College of Medical Sciences' 2008 graduating class, residing across three continents. This program aimed to facilitate research collaboration and skill development among colleagues with diverse research experience levels, fostering a supportive environment for career development in research. The group relied heavily on digital technology to carry out its activities due to the different geographical locations of the group members. Led by experienced peer leaders, the group fostered a collaborative learning environment where members leveraged each other's expertise. Within 18 months, we published two research papers in high-impact peer-reviewed global health journals, launched a mixed-methods research study, and conducted training sessions on research design and implementation. Findings from our work were presented at conferences and workshops. However, logistical hurdles, competing priorities, structural constraints, and uneven participation presented challenges.

**Conclusion:**

The peer mentorship collaboration has achieved some successes so far, and this model can be emulated by other cohorts of medical professionals across LMICs. Despite the group's success at a micro- or individual level, there remain significant structural barriers to research capacity building in LMICs that can only be addressed at the meso- and macro-levels by institutions and government, respectively. A systems-level approach is required to develop and support research capacity building and foster global research collaboration and effectively turn brain drain into brain gain.

## Background

Africa, which contributes about 18% of the world's population [1], accounts for only 1.3% of the world's annual research output, with just three countries—Kenya, Nigeria, and South Africa—contributing 52% of Africa's total output [2]. Building and retaining human resources for health (HRH) research capacity is essential for improving the quality and effectiveness of healthcare systems in Africa's low and middle-income countries (LMICs) [2–4]. Unfortunately, research capacity development in these settings is limited and fraught with numerous challenges, including inadequate training in research methodologies, lack of mentorship and institutional support in conducting research, and lack of research funding opportunities^,^ [4–8] Indeed, this lack of availability of resources and support for a career in research in their local institutions is one of the key drivers for the migration of health workers to high-income countries (HICs), where such opportunities abound [9]. The migration of these highly trained individuals, aptly coined "brain drain" causes a loss of intellectual and technical resources for LMICs [10], and has reached alarming levels in recent years. As of 2021, less than half of the registered doctors in Nigeria were practicing in the country, as many of whom had migrated to HICs in search of better career prospects. [11]

Mentoring fosters professional development and facilitates collaborations, particularly in academic medicine [12]. Frazen et al. (2016) found that peer mentorship was a popular strategy proposed to address the barriers to research in LMICs in their systematic review of major approaches to health research capacity development [6]. While traditional mentoring approaches are widely recognized, alternative models like peer mentoring have recently gained attention and investigation. In peer-mentoring programs, individuals of similar academic rank and interests collaborate within a facilitated framework, sometimes guided by a faculty member of higher academic standing, to collectively pursue their scholarly goals [13]. Peer mentoring can be a legitimate means through which academics from LMICs who have emigrated to HICs to collaborate with their colleagues back in their source countries on important public health research that affects the global population, given their understanding of both contexts [14, 15]. Peer mentoring can aid the transfer of knowledge from the mentors to mentees, of various skillsets such as developing research skills, writing academic papers and grants, navigating the academic publishing process, networking with other researchers, and securing fellowship funding opportunities [14]. Peer mentoring, thus, offers an opportunity to convert "brain-drain" to resource profit or "brain gain" for LMICs [10], by strengthening research capacity in LMICs through these collaborations.

In recent years, Nigeria has witnessed a significant exodus of highly skilled health workers, including doctors and dentists from the 2008 graduating class (colloquially called the Diamante graduating class) of the University of Benin, to pursue better career opportunities and improved living standards abroad. This alarming trend not only jeopardizes the quality of healthcare services back home, but also highlights the urgent need to strengthen health research capacity within the country. The inadequate research capacity among clinicians, not just in Nigeria but globally, is a pressing issue that demands immediate attention. This concern is particularly acute given the increasing prominence of research in shaping effective healthcare delivery and leadership [3]. As members of the Diamante graduating class ascend to key leadership positions within the Nigerian healthcare system, a noticeable divide might emerge, separating those trained solely in core clinical skills from those who have become additionally equipped with strong global health research capabilities. This unique opportunity necessitates a strategic intervention to harness the skillset of the latter group, enabling them to contribute their expertise to the development of the former group. We present a case study of a peer-mentoring program for doctors who graduated from the University of Benin College of Medical Sciences in 2008. This case study explores the potential of peer mentorship to tackle two critical issues: bridging health research capacity of doctors from LMICs and the transformation of human resource for health brain drain into "brain gain".

## Approach

The "Diamante Research Group" was created in 2021 as a subset of the University of Benin College of Medical Sciences Medical and Dental graduating class of 2008. The group was formed to foster research collaboration among graduates who were passionate about pursuing a research career, but had different levels of experience and skills in conducting research. The overarching aim of the group was to leverage the research expertise of colleagues with extensive training and experience to enhance the research capabilities of interested clinicians who lacked the necessary support or experience.

**Fig. 1:**
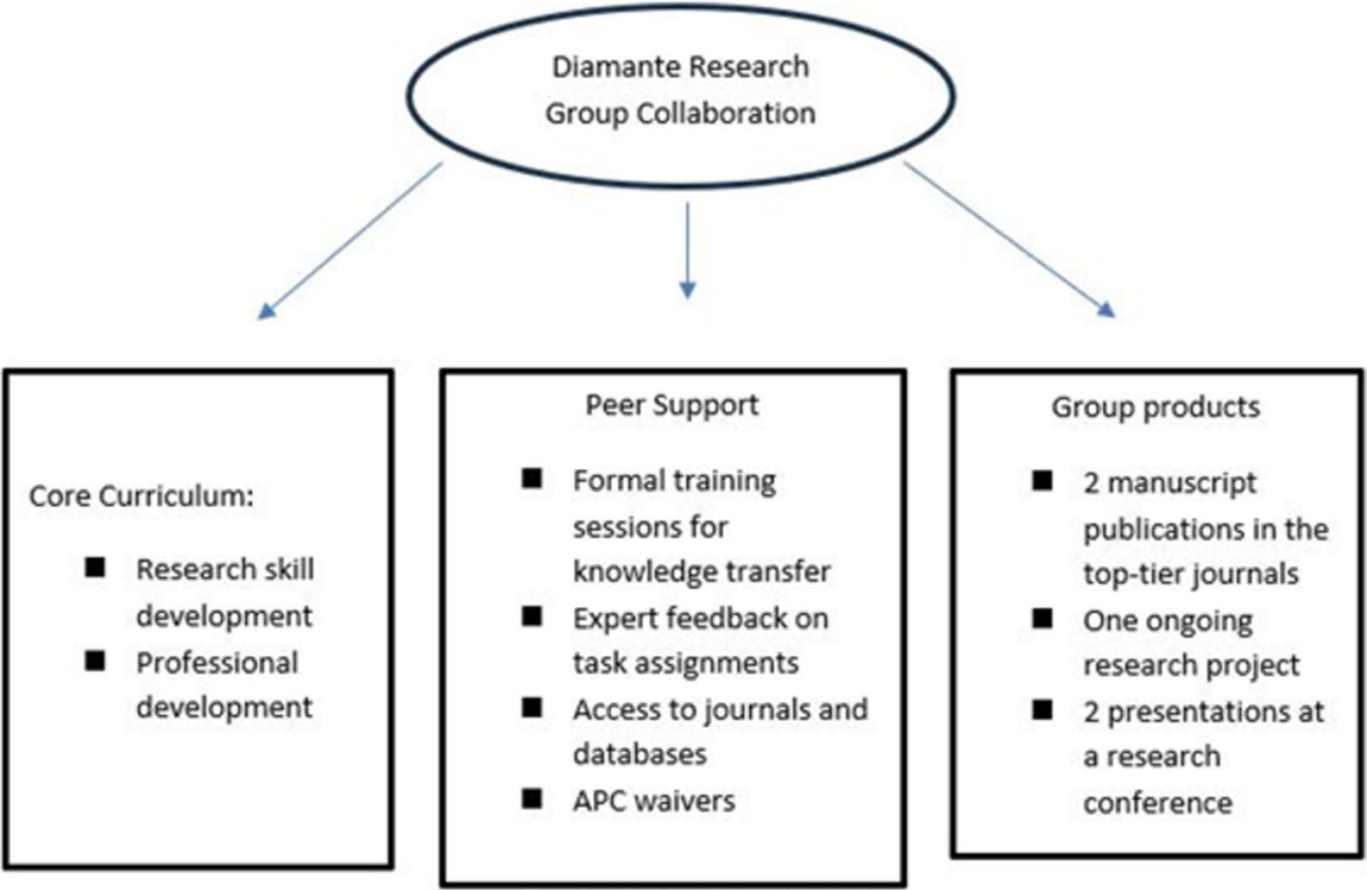
Diamante Research Group peer mentoring model adapted from the iMeRGE peer mentoring model by Bussey-Jones et al. [16]

The conceptual framework guiding the formation and activities of the Diamante Research Group is comparable theoretically to the peer mentorship model developed by the Internal Medicine Research Group at Emory (IMeRGE) group to build the research and academic skills of the group members and ensure their career advancement in academic medicine (see Fig. 1) [16]. Unlike the IMeRGE group, however, the Diamante Research Group members were location in different institutions in different countries, spanning three continents, including Africa, Europe and North America. Hence, the group relied on digital technology to carry out its activities.

### Group structure

The group started with 16 doctors in diverse clinical and non-clinical areas of specialization, including pediatrics, oral medicine, obstetrics and gynecology, orthopedics, and research. The group members, consisting of six women and ten men, resided in four countries: Canada, Nigeria, the United Kingdom, and the United States of America. Three members were selected to co-lead the group and facilitate its activities to achieve the outlined goals and objectives. These group leaders were chosen based on their research experience and training. Two of them were well-published and had previously and currently led research projects. They had pursued additional research training in the United Kingdom and the United States of America, ultimately earning PhDs after completing rigorous research programs. The third group leader was in the process of obtaining a master’s degree in health policy, was still resident in Nigeria, thus provided contextual and local support. Additionally, several other group members had either acquired or were enrolled in master’s degrees in public health in Nigeria or elsewhere. Table 1 shows the details of the initial group members.

**Table 1:** Demographic information of group members (*N* = 16), Diamante Research Group

Category	Total number (percent)
Respondents	
Female	6 (37.5)
Male	10 (62.5)
Degrees attained or in process	
BDS*	5 (31.3)
MBBS*	11(68.7)
PhD	2 (12.5)
Master's degree	10 (62.5)
Area of clinical specialization (i.e., postgraduate clinical specialization)	
Community Dentistry	1 (6.3)
Obstetrics and Gynecology	1 (6.3)
Oral Medicine	2 (12.5)
Oral Pathology	1 (6.3)
Orthopedic Surgery	1 (6.3)
Pediatrics	4 (25.0)
Preventive Dentistry	1 (6.3)
Restorative Dentistry	1 (6.3)
None	4 (25.0)
Country of residence	
Canada	2 (12.5)
Nigeria	11(68.7)
United Kingdom	1(6.3)
United States	2 (12.5)
Employment sector (respondents may work in multiple sectors)	
Academia	13 (81.3)
Clinical practice	11(68.7)
Government	12 (75.0)
Non-government organization	2 (12.5)

*All members of the group either had an MBBS (Bachelor of Medicine, Bachelor of Surgery) or a BDS (Bachelor of Dental Surgery) as they had all graduated from University of Benin College of Medical Sciences in 2008. Respondents may have additional degrees hence the total percentage for this section is greater than 100%

### Group activities

The inaugural meeting of the Diamante Research Group was held in December 2021. At this meeting, the objectives and expectations were established through a priority-setting approach involving all group members, to guide activities from the outset. The group objectives fell into three main categories (see Fig. 1):

1. *Skills acquisition.* Increasing research skills and knowledge via didactic sessions on topics, such as conducting systematic reviews, grant-writing, research design and methodologies, and publication ethics, etc.
2. *Access to resources.* Increasing access to research tools, resources, and opportunities such as access to grant information, access to journals and databases through academic network and membership subscriptions, access to databases, access to academic librarian expertise for research projects.
3. *Skills application.* Conducting research projects together to provide hands-on experience in all stages of research, from developing a research question to submission of a completed project for publication, guided by international best practices in research and publication ethics.

Leveraging digital technology, the group addressed the challenges posed by members being in different locations and time zones. WhatsApp, Zoom, and email were essential for coordinating meetings and events, with all gatherings held virtually via Zoom. These Zoom meetings occurred every two to four weeks, depending on the demands of ongoing research projects or key milestones. Additional modes of correspondence included regular email exchanges, updates on the Diamante Research Group WhatsApp platform, and phone calls between members. Minutes of the Zoom meetings were shared on the WhatsApp platform. The group leaders maintained a dedicated WhatsApp platform for ongoing engagement. Additionally, the group created a secure folder on Google Drive to store documents related to group projects, accessible to all members.

For each research project, the group created task-sharing documents detailing all tasks involved in the various project stages. Members volunteered for tasks based on their interests and perceived competencies. Each task was led by a mini-team head, who was supervised by the main group leaders. For some tasks, the main group leaders provided direct supervision due to their technical nature. The group also used Gantt charts to create timelines and track the progress of each project. To ensure fairness and adhere to ethical publication standards regarding authorship, we tracked participation in group projects using an authorship tool guide.

## Outcome

The main goal in the formation of the Diamante Research Group, like similar peer-mentorship research groups, was to collaboratively work on research projects that would lead to tangible outcomes, such as publications, conference abstracts, and grant funding [5, 14, 16].

*Skills acquisition and access to resources.* The group conducted real-time virtual lecture series on systematic reviews, including guided practice sessions on tasks such as formulating review questions, conducting database searches, exporting search outputs, and screening articles using collaborative platforms like Rayyan®. Additionally, the group developed search strategies, conducted searches, and exported search results for the main systematic review project via Zoom. This approach provided members with hands-on experience, reinforcing the knowledge gained from the lecture series. The group leveraged the institutional access of its leaders to obtain access to databases and journal articles that would have otherwise required significant monetary commitment, especially for those still residing in Nigeria [17].

*Skills application.* Within the first 18 months, the group had published two research papers in high-impact, peer-reviewed global health journals and commenced a primary mixed-methods study. The inaugural project undertaken by the group was a systematic review on the drivers of migration of health workers from LMICs. The group collectively prioritized conducting this systematic review, which subsequently laid the foundation for the group's ongoing primary mixed-methods cohort study. Before starting the review, the group registered the protocol in an open-access online database of systematic review protocols. The review protocol and the subsequent research article were both published in British Medical Journals (BMJ Open and BMJ Global Health) [9, 18]. The group successfully obtained waivers for the Article Processing Charges (APC) for both journal publications. High APCs are a significant barrier, often excluding researchers from LMICs, especially those without research fundings and perpetuating the under-representation of their voices in high-impact journals. [17]

*Dissemination of research.* The group members presented their research at the Inaugural University of Benin College of Medicine Scientific Conference, themed “Medical Education and Brain Drain: Implications for Health Workforce Development in Nigeria”, held in August 2023 at their alma mater.

Throughout these processes, the group leaders mentored their peers, leveraging their wealth of experience to guide them on how to navigate the process, fostering a shared learning approach. The transfer of knowledge on research skills from more experienced colleagues in the diaspora to those with less experience and access to research tools, and resources, that would have otherwise been unavailable, represent some of the ways the group transformed brain drain to brain gain.

At present, we have completed the data collection for our cohort study and are in the process of writing up the findings for submission for the third publication by the group. In addition, we are conducting training sessions on designing and implementing qualitative and mixed-methods studies in preparation for our next research project. The goal for the next year is to focus on training on grant-writing with practical applications in the form of applying for and hopefully, securing grant funding to carry out future research projects on a larger scale. Lastly, an additional eight colleagues from the 2008 graduating class have requested to join the research group following the success of the initial projects, bringing the number of group members from the initial 16 to a total of 24 members.

## Challenges and reflections

We did encounter several challenges as a peer mentoring group (Table 2), similar to other peer mentorship programs reported in the literature [14, 16]. Firstly, the significant differences in time zones due to the location of the members made it difficult to schedule meetings at a time that was convenient for all group members. In addition, other professional and personal responsibilities of the members such as on-call duties and caring for their young children also deterred attendance of or full participation during group meetings [16]. Unstable Wi-Fi signal, especially for our peers in Nigeria also interfered with the quality of the Zoom meetings, further highlighting critical infrastructure issues that hamper research in LMICs [14]. To address these challenges, we scheduled meetings on weekends at a consistent day and time that was convenient for most of the group. Meeting dates were set in advance, and multiple reminders were sent beforehand. The group leaders shared the agenda ahead of time, and one of them facilitated the meetings to ensure efficient use of time. After each meeting, the minutes and action items were shared on the WhatsApp group to keep absent members informed.

**Table 2:** Summary of challenges encountered by the Diamante peer mentorship research group and team responses to them

Broad challenges	Specific challenges	Response
Logistics challenge	Different locations and time zones of members affected the scheduling of meetings	• Selected a specific day and time of the week that suited the majority of members and scheduled meetings on the same day and time of the week • Scheduled meetings ahead of time to aid planning for attendance
Competing responsibilities	Childcare responsibilities and clinical on-call duties interfered with meeting participation	• Acknowledged and supported the importance of work–life balance for members and excused members from meetings as needed • Required all members to mute their microphones while attending meetings to prevent disruption from background activities • Provided summaries of meetings and next steps on group chats to catch up members who could not attend meetings
Structural infrastructure challenges	Unstable Wi-Fi, internet access in and electricity supply in Nigeria acted as barriers to meeting attendance and participation	• Provided summaries of meetings and next steps on group chats to catch up members who could not attend meetings
Inconsistency in member participation	Incomplete or poorly completed tasks	• Set clear expectations of the deliverables on each task a priori • Utilized an authorship guide tool to track task completion and member contribution in a transparent manner

We also encountered challenges related to inconsistencies in the quality of work performed by different group members. These variations stemmed from factors such as insufficient skills to complete assignments, inadequate commitment to tasks, or a combination of both factors. Unlike in traditional mentor–mentee relationships, where power dynamics often drive performance, our peer-group structure lacked such pressures, resulting in less stringent adherence to expectations [16]. Upon identifying these issues early in the project, the group leaders decided it was crucial to establish clear, predefined criteria for what constituted acceptable completion of each task and to communicate these expectations upfront going forward using an authorship guide tool.

Points on the authorship guide tool were awarded only upon satisfactory completion of a task. It also became necessary to limit the number of volunteers for specific tasks and, in certain cases, select group members based on their demonstrated proficiency and commitment in previous tasks. The authorship guide tool was regularly updated and shared as each project progressed to ensure transparency and accountability. Members were encouraged to discuss any concerns about the points allocated to them with group leaders, and these issues were addressed individually. Ultimately, establishing clear expectations and maintaining a transparent, albeit imperfect, system for tracking member contributions has proven effective in promoting fairness and accountability within the group.


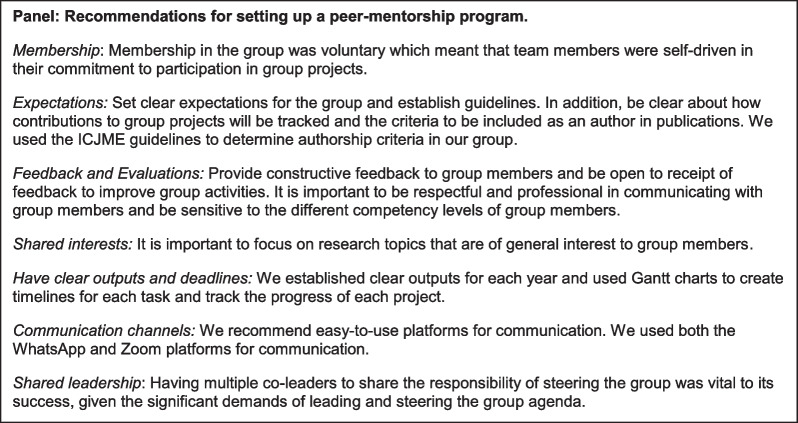


We have established a successful peer mentoring group which could be emulated by other cohorts of medical professionals across LMICs, and we provide several actionable recommendations in setting up similar programs for interested parties (see panel). However, we note that there remain significant structural barriers to research capacity building that can only be addressed at the meso- and macro-levels such as protected research time and funding for research projects. A systems-level approach led by institutions and government is required to develop and support research capacity building and foster global research collaboration to effectively turn brain drain into brain gain.

## Conclusion

The peer mentorship group has proven beneficial to all participants involved, offering members the opportunity to collaborate on research topics of mutual interest. During the group audit following the successful completion of our initial project, participants expressed satisfaction with the group's achievements and noted an increase in their research knowledge and skills essential for academic success. Through teaching and mentoring their peers, group leaders enhanced their own research leadership abilities and gained valuable experience in guiding teams to project completion.

Moreover, personal connections have flourished among members, leading to planned future research collaborations beyond the primary group among those sharing similar interests. Peer mentorship plays a critical role in professional development and career advancement, potentially bridging gaps in access to expertise, funding, research tools, and opportunities between academic institutions in high-, middle-, and low-income countries. Ultimately, this approach aims to transform brain drain into brain gain.

## Data Availability

There is no data underlying this manuscript.

## Abbreviations

HRH: Human Resources for Health
LMICs: Low- and middle-income countries
HICs: High-income countries
IMeRGE: Internal Medicine Research Group at Emory
APC: Article processing charges

## Declarations

### Patient and public involvement

Patients and/or the public were not involved in the design, conduct, reporting, or dissemination plans of this research.
